# Improving the Volume Stability of Low-Carbon Ultra-High Performance Concrete Through the Employment of Internal Curing

**DOI:** 10.3390/ma19143100

**Published:** 2026-07-19

**Authors:** Yongquan Zhang, Yanyan Zhou, Asigen, Jinshan Yu, Meiqi Cao, Mandula

**Affiliations:** 1 Transportation Institute, Inner Mongolia University, Hohhot 010021, China; 18746364247@163.com (Y.Z.); caomq890619@163.com (M.C.); 2 Inner Mongolia Transportation Design and Research Institute Co., Ltd., Hohhot 010010, China; 13084716893@163.com; 3 School of Transportation and Municipal Engineering, Inner Mongolia Technical University of Construction, Hohhot 010070, China

**Keywords:** ultra-high performance concrete, high volume fly ash, aeolian sand, internal curing, volume stability

## Abstract

**Highlights:**

- The addition of SAP significantly inhibited the shrinkage and early-age cracking of UHPC.
- The reduction in compressive strength of UHPC was negligible when 0.3% SAP with a particle size range of 180–600 μm was used.
- The incorporation of SAP improved the microstructure compactness and refined the pore structure by providing internal curing.

**Abstract:**

High-volume fly ash binder and aeolian sand have been widely used in the production of low-carbon ultra-high performance concrete (UHPC) owing to their environmental and economic benefits. However, such low-carbon UHPC still faces the volume stability issues, including autogenous shrinkage, drying shrinkage and early-age cracking. In this study, a superabsorbent polymer (SAP) was incorporated into low-carbon UHPC containing high-volume fly ash and aeolian sand to improve its volume stability through internal curing. The effects of SAP particle size and dosage on the mechanical properties and volume stability of low-carbon UHPC were investigated. Results showed that the addition of 0.3% SAP with a particle size of 180–600 μm significantly reduced shrinkage and early-age cracking while maintaining the compressive strength. Furthermore, the microstructure of low-carbon UHPC was characterized using advanced techniques to shed light on the underlying mechanisms.

## 1. Introduction

Ultra-high performance concrete (UHPC) is characterized by its exceptional mechanical property, particularly its compressive strengths which can be as high as 120 MPa, as well as the improved durability [1,2]. This unique combination makes it promising for various construction applications, including roadways and bridges [3,4]. Normally, the outstanding performance of UHPC is achieved through the application of a high content of cement and a low water-to-cement ratio and the inclusion of steel fibers. In order to reduce the carbon dioxide emission associated with the substantial cement content in UHPC, the supplementary cementitious materials, including silica fume, fly ash, ground granulated blast furnace slag, metakaolin and glass powder, have been used to partially replace cement during the preparation of UHPC [5,6,7]. At a high level of replacement, fly ash has been widely investigated for its effect on the workability, mechanical properties, and durability of UHPC [8,9,10]. For instance, a maximum compressive strength of 106.9 Mpa can be achieved with 70% replacement of cement by fly ash in UHPC.

Recently, aeolian sand has been used to replace the conventional fine aggregate during the preparation of UHPC to further reduce its embodied carbon emission, especially in desert regions [11]. Particularly, aeolian sand can be used to produce UHPC using an enhanced Andreasen and Andersen particle packing model [12]. Moreover, the compressive strength of UHPC can be as high as 207.0 Mpa with entirely replacing river sand with aeolian sand [13].

Although UHPC features numerous advantages, its low water-to-binder ratio can cause a decrease in the internal relative humidity, resulting in an increased shrinkage of UHPC [14,15]. This becomes more deleterious when UHPC is exposed to restrained conditions and conventional curing environments. However, owing to the dense microstructure and low permeability of UHPC, external water curing is normally ineffective, necessitating the employment of internal curing [16,17]. Among the materials that can provide internal curing in UHPC (e.g., highly absorbent resins, porous aggregates, or ultrafine powders), superabsorbent polymer (SAP) is the most prevalent [18]. It was reported that the addition of SAP at an appropriate dosage can completely eliminate the autogenous shrinkage of UHPC [19]. On the other hand, it should be noted that the presence of an excessive amount of SAP adversely affects the mechanical properties of UHPC, even though the shrinkage-induced cracking can be effectively controlled [20].

However, the application of internal curing has been investigated primarily for conventional UHPC with relatively high cement contents and conventional fine aggregates [14,20,21,22]. The effectiveness of SAP in low-carbon UHPC incorporating high-volume fly ash and aeolian sand remains insufficiently understood. Compared with conventional UHPC, the incorporation of high-volume fly ash significantly alters the hydration kinetics and internal water demand, while aeolian sand exhibits distinct particle characteristics that may influence the pore structure and moisture transport. Consequently, the internal curing mechanisms and the resulting volume stability of such low-carbon UHPC may differ substantially from those reported for conventional UHPC.

To address this research gap, this study investigated the effects of SAP particle size and dosage on the internal relative humidity, autogenous shrinkage, drying shrinkage, early-age cracking behavior, and mechanical properties of low-carbon UHPC containing high-volume fly ash and aeolian sand. Furthermore, X-ray diffraction (XRD), thermogravimetric analysis (TG), mercury intrusion porosimetry (MIP), and scanning electron microscopy (SEM) were employed to shed light on the mechanisms by which SAP improved the volume stability of this sustainable UHPC system.

## 2. Materials and Methods

### 2.1. Materials

The cementitious materials used to prepare UHPC comprised ordinary Portland cement (P·O 52.5R), fly ash, ground granulated blast furnace slag and silica fume. Table 1 shows the chemical compositions of these materials. The aeolian sand was derived from Chifeng region of Inner Mongolia, featuring particle size ranging from 75 to 600 μm. A polycarboxylate-based superplasticizer with a solid content of 39% was used as water reducer. Copper-coated hooked steel fiber measuring 0.2 mm in diameter and 12 mm in length were utilized. Three types of SAP with different particle size range, including 180–600 μm (SL), 150–250 μm (SM) and 125–165 μm (SS), were used to provide internal curing for UHPC. The selection of these size ranges was based on recent studies [23,24], which indicated that the particle size of SAP significantly influenced water absorption–desorption kinetics of UHPC.

**Figure 1:**
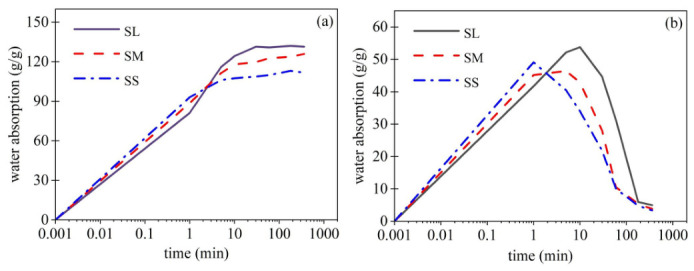
Water absorption of SAP in (**a**) water and (**b**) cementitious filtrate.

The water absorption behavior of the three SAPs was evaluated in both tap water and cementitious using the tea bag method. To prepare the cementitious filtrate, a slurry with the same cementitious composition as the UHPC mixtures and a water-to-binder ratio (w/b) of 0.5 was first prepared and subsequently filtered. The water absorption behavior of the SAPs in both media is presented in Figure 1. As shown in Figure 1, all three SAPs exhibited progressive water absorption over the 6 h test period in tap water, whereas distinctly different behavior was observed in the cementitious filtrate. In tap water, the absorption rate decreased with increasing particle size, indicating that larger SAP particles required a longer time to reach equilibrium because of the longer diffusion path for water penetration. In contrast, all three SAPs rapidly reached their maximum absorption within the first 10 min in the cementitious filtrate, followed by a gradual decrease in the absorbed water content. This behavior can be attributed to the high ionic concentration of the cementitious pore solution, which limited the swelling capacity of SAP and promoted the gradual release of the absorbed solution as hydration proceeded. Such abruption–desorption behavior enabled SAP to function as an internal water reservoir, supplying water to the surrounding cementitious matrix during hydration and thereby mitigating self-desiccation and autogenous shrinkage.

### 2.2. Mix Proportion and Specimen Preparation

#### 2.2.1. Mix Proportion

Table 2 lists the detailed mix proportions of samples in this study. Three different particle sizes of SAP at the dosage of 0.3% and 0.6% by mass of cementitious materials, respectively, were introduced into low-carbon UHPC, resulting in a total of six UHPC mixtures containing SAP. These dosage levels were chosen based on previous studies [20,22], which demonstrated that 0.3–0.6% SAP can effectively mitigate the autogenous shrinkage of UHPC while maintaining acceptable mechanical performance. The SAP particles were pre-saturated with water before mixing. The water absorption capacity used for mixture design was approximately 40 g/g, resulting in additional internal curing water corresponding to w/b increments of 0.012 and 0.024 for SAP dosages of 0.3% and 0.6%, respectively. The effective w/b utilized in these UHPC mixtures was 0.16, leading to the total w/b being 0.172 and 0.184 when considering the pre-absorbed water contained in SAP at the dosages of 0.3% and 0.6%, respectively.

The dosage of the polycarboxylate superplasticizer was increased for the mixtures containing 0.6% SAP in order to compensate for the reduction in workability caused by the higher SAP dosage and to ensure adequate mixing and casting quality. The adjustment was made solely to maintain the workability of the fresh mixtures and did not alter the effective w/b ratio. In addition, three reference UHPC mixtures with w/b ratios of 0.16, 0.172 and 0.184 were also prepared as a control. It should be noted that the content of fly ash in all UHPC mixtures was kept at 50% by the mass of cementitious materials, while the aeolian sand-to-cementitious materials ratio was 1 among these mixtures. The content of steel fiber in all samples was kept constant at 2 vol% of the cementitious materials.

Compared with conventional UHPC mixtures reported in the literature, which typically contain about 800 kg/m^3^ cement, 200 kg/m^3^ silica fume and 1000 kg/m^3^ quartz sand, the proposed mixture reduced cement consumption by approximately 63%. A simplified embodied carbon estimation was further conducted using typical emission factor from the literature (0.85 kg CO_2_/kg for cement, 0.02 kg CO_2_/kg for fly ash, 0.03 kg CO_2_/kg for silica fume, 0.06 kg CO_2_/kg for slag, 0.005 kg CO_2_/kg for aeolian sand, and 0.01 kg CO_2_/kg for quartz sand). The estimated CO_2_ emission of the proposed UHPC was approximately 279 kg CO_2_/m^3^, whereas a conventional UHPC containing 800 kg/m^3^ cement, 200 kg/m^3^ silica fume and 1000 kg/m^3^ quartz sand would generate approximately 696 kg CO_2_/m^3^. This corresponded to an estimated reduction of about 60% in CO_2_ emission. In this case, the term “low-carbon UHPC” referred to the substantial reduction in cement consumption and associated embodied carbon emissions.

#### 2.2.2. Specimen Preparation

The cementitious materials, aeolian sand and pre-soaked SAP were firstly mixed for 1 min at 60 rpm. Subsequently, water and superplasticizer were mixed and added into the dry materials for another 3 min mixing at the same mixing speed, which was followed by a further mixing for 1 min at an increased mixing speed of 120 rpm. At last, steel fiber was gradually added into the fresh mixture and mixed for 3 min. After mixing, the fresh mixture was cast into molds for curing in a standard curing chamber (relative humidity > 95%, temperature kept at 20 ± 2 °C) for 24 h. The samples were transferred to water curing after demolding. Three replicates for each UHPC mixture were used to evaluate the mechanical properties and volume stability, while the experimental results were reported as the mean values with their corresponding standard deviations.

### 2.3. Experimental Methods

#### 2.3.1. Mechanical Properties and Volume Stability

Due to the alteration of rheological behavior of cement-based materials by the presence of SAP, which can reduce free water and increase interparticle friction [23,25], the flowability of the fresh mixture was carried out according to the Chinese standard GB/T 2419–2005 [26] after the mixing was completed.

Compressive strength was assessed following the guidelines outlined in the Chinese standard GB/T 17671-2021 [27]. Prism-shaped UHPC samples with size of 40 mm × 40 mm × 160 mm were employed for the compressive strength test. Prior to the compressive test, the prisms were split into two halves using a three-point loading configuration. The compressive strength test was conducted using the split samples.

**Figure 2:**
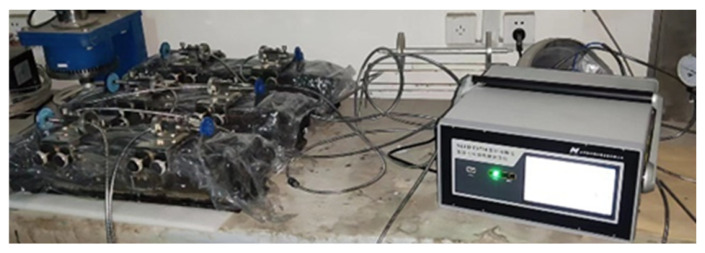
The experimental apparatus for testing autogenous shrinkage of UHPC.

According to the Chinese standard GB/T 50082-2019 [28], autogenous shrinkage of UHPC was measured using prismatic samples with size of 100 mm ×100 mm × 515 mm. After casting the fresh UHPC mixture into the pre-lubricated mold, the immediate sealing was applied by coating the top surface with two layers of plastic film. The samples were stored at the ambient temperature of 20 ± 1 °C. The autogenous shrinkage data were recorded from the twelfth hours after finishing the casting (around the initial setting time) and then collected automatically every 15 min, using a non-contact shrinkage measurement instrument as shown in Figure 2.

**Figure 3:**
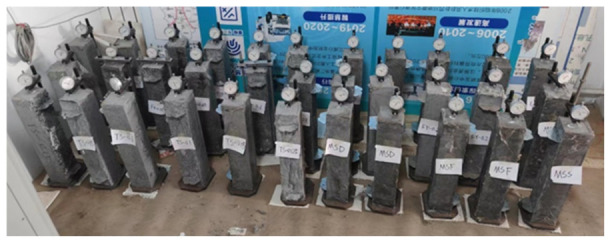
The experimental apparatus for testing drying shrinkage of UHPC.

After finishing the autogenous shrinkage test, the samples were demolded and transferred to the drying shrinkage testing apparatus as specified in the Chinese standard GB/T 50082-2019, as presented in Figure 3. During the test, the samples were stored in an environment with temperature and relative humidity of 20 ± 1 °C and 50 ± 5%, respectively. The measurement was recorded daily using a 0.001 mm dial gauge over a 40-day period.

The internal relative humidity was monitored by an embedded sensor in the sealed cubic sample (100 mm × 100 mm × 100 mm). During the casting process, a stainless-steel rod with its attached PVC tube was initially placed at 50 mm beneath the top surface of the sample. When the mixture began to set, the stainless-steel rod was removed from the PVC tube, while the sensor was promptly inserted into the tube, enabling the lower part of the sensor effectively monitoring the humidity changes in the adjacent mixture [29]. Two rubber O-rings were attached to the upper part of the sensor to prevent the ingress of air. At last, the sample with its embedded sensor was sealed using multilayer plastic film and epoxy resin, which was placed in a controlled environment chamber with temperature and relative humidity maintained at 23 ± 1 °C and 50 ± 3%, respectively. The measured data were collected by the sensor at 12 h intervals over a span of 7 days.

The early-age crack test was conducted using 320 mm × 240 mm × 40 mm samples. The four edges of the sample were restrained using 14 equally spaced steel screws on each side, which prevented free shrinkage and generated restrained tensile stresses during drying. After casting, the samples were placed in an environment, in which the temperature and relative humidity were maintained at 20 ± 1 °C and 50 ± 5%, respectively. In order to accelerate the initiation of cracking, a fan was positioned approximately 300 mm above the sample surface and directed vertically downward to provide a uniform airflow across the exposed surface. The airflow condition was controlled by maintaining a constant air velocity of approximately 5 m/s throughout the 24 h exposure period. At the end of the test, the width of cracks was measured using a 100× optical microscope, whereas the crack length was determined using a steel ruler. The crack area per unit area was calculated from the measured crack width and length of all visible cracks. Three samples were prepared and tested for each mixture, and the reported crack parameters represent the average values.

#### 2.3.2. Microscopic Characterization

The hydration products of UHPC with varying dosages of SAP were characterized using an X-ray diffractometer (Rigaku SmartLab SE, Tokyo, Japan). At specific curing ages, UHPC samples were soaked in ethyl alcohol and subsequently dried at 60 °C to stop further hydration process. Following this, the samples were ground into powder with size smaller than 75 μm. XRD analysis was performed using a scanning rate of 5 °/min with Cu Kα radiation in the range of 10 to 80°.

TG analysis was conducted to characterize the hydration process of UHPC samples using a thermogravimetric analyzer (HITACHI STA200, TA Instrument, Tokyo, Japan). Similar with that used in the XRD test, the samples were also soaked in ethyl alcohol and dried at the designed curing time to stop hydration. Subsequently, the samples were ground into powder and subjected to the heating process, which was conducted under a nitrogen atmosphere at a heating rate of 10 °C/min until reaching 1000 °C.

MIP test was performed to investigate the pore structure of UHPC samples. An Auto Pore V-9600 instrument (Micromeritics Instrument Corporation, Norcross, GA, USA), applying a high pressure of 228 MPa and a contact angle of 130°, was utilized for the analysis. After curing for 3, 28 and 180 days, the hydration cessation and subsequent drying were applied to samples before they were subjected to MIP analysis.

For SEM observation, small samples were extracted from the crushed UHPC mixture and immersed in ethanol for 48 h to halt further hydration, followed by drying at 60 °C. Subsequently, grinding and polishing were performed to ensure a smooth surface quality. UHPC samples were coated with gold before they were subjected to the scanning electron microscope (TESCAN MIRA LMS, TESCAN, Brno, Czech Republic). The equipped energy-dispersive X-ray spectroscopy (EDS) (Oxford Instruments, Abingdon, UK) can also be used to analyze the chemical composition of hydration products.

## 3. Results and Discussion

### 3.1. Flowability

**Figure 4:**
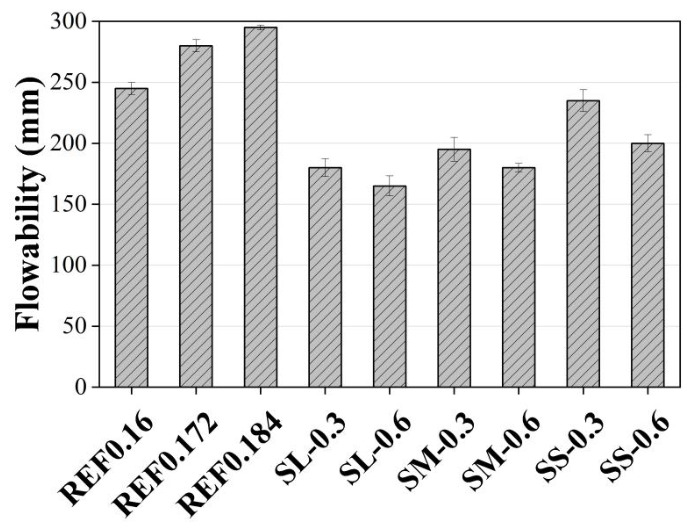
Influence of particle size and dosage of SAP on the flowability of low-carbon UHPC.

Figure 4 shows the effects of SAP particle size and dosage on the flowability of UHPC. It can be seen from this figure that compared to reference UHPC without SAP, the incorporation of 0.3% and 0.6% SAP can result in a 4.1–32.6% reduction in flowability of fresh UHPC mixtures. Moreover, the flowability of UHPC declined as the dosage of SAP increased. This reduction is due to the water sequestration and rheological disturbance induced by SAP. Pre-saturated SAP retains a fraction of the mixing water, reducing the free water available for particle lubrication and effectively increasing the plastic viscosity of the fresh mixture [23,25]. In addition, the swollen SAP behaves as soft, irregular inclusions that disrupt the optimized particle packing and increase the interparticle friction, further hindering the flowability of UHPC [25,30]. As a result, a greater reduction in flowability of UHPC can be observed with increasing the dosage of SAP.

Compared with the corresponding control mixtures that have the same total w/b ratio (REF0.172 and REF0.184), the SAP-containing mixtures exhibited lower flowability. This result confirmed that the additional water stored in SAP did not contribute to the workability of the fresh mixture in the same manner as free mixing water, but instead remained temporarily immobilized and was subsequently released during hydration to provide internal curing. It should be noted that a higher dosage of superplasticizer was used in the mixtures containing 0.6% SAP to achieve acceptable workability. Although the increase in superplasticizer may have partially compensated for the loss of flowability, the 0.6% SAP mixtures still exhibited lower flowability than the corresponding control mixtures. Therefore, the observed reduction in workability can primarily be attributed to the incorporation of SAP rather than to differences in superplasticizer dosage.

### 3.2. Compressive Strength

**Figure 5:**
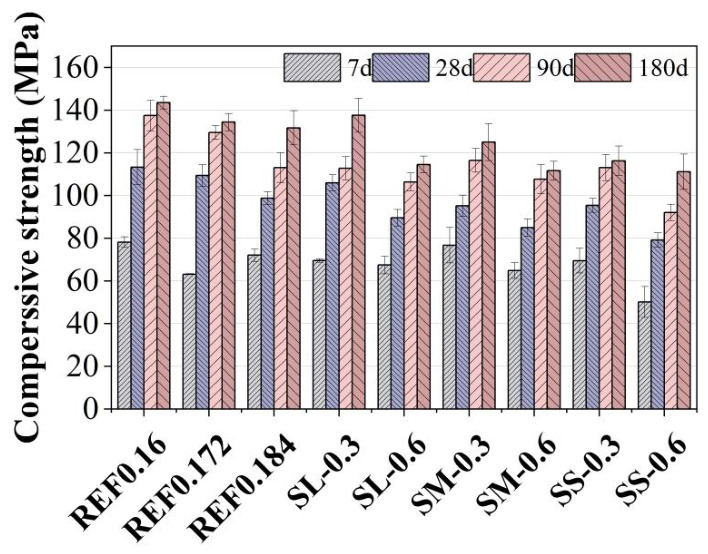
Influence of particle size and dosage of SAP on the compressive strength of low-carbon UHPC.

Figure 5 illustrates the variation in compressive strength of UHPC with different particle sizes and dosages of SAP over a 180-day curing period. The result indicates that the addition of SAP adversely affected the compressive strength of UHPC. This is consistent with findings from recent studies [22,30], which reported that SAP-induced voids and delayed water release can lead to early strength reduction, although the long-term strength can be partially recovered due to the continued hydration. Specifically, the strength loss caused by the larger dosage of SAP is more significant than that of the smaller dosage. For instance, when 0.6% of SAP with a particle size of 150–250 μm was added and the total w/b remains unchanged, the compressive strength of UHPC at the curing time of 7 and 180 days decreased by 9.9% and 15.3%, respectively, compared to the control group REF0.184. This phenomenon can be attributed to the formation of SAP-induced voids after water released during hydration [14]. Although the gradual release of internally stored water promoted continued hydration, the increase in pore volume associated with a higher SAP dosage outweighed the beneficial internal curing effect, resulting in a net reduction in compressive strength. The comparison between the SAP-containing mixtures and the corresponding control mixtures with identical total w/b ratios demonstrated that the additional water stored in SAP functioned as internal curing water rather than free mixing water. Consequently, maintaining a low effective w/b ratio while providing internal curing was more effective for preserving the compressive strength of UHPC than simply increasing the total water content.

On the other hand, SAP with smaller particle size exhibited a more significant negative impact on the compressive strength of UHPC than larger SAP at the same dosage. For instance, at the curing time of 180 days, the compressive strength of UHPC containing 0.3% SAP with particle size ranges of 180–600 μm, 150–250 μm and 125–165 μm decreased by 4.1%, 13.1% and 22.6%, respectively, compared with the control group REF0.16. Similar observations have also been reported in previous study [31]. It has been suggested that the influence of SAP particle size on the mechanical properties of cementitious materials was governed by a combination of factors, including the absorption/desorption characteristics of SAP, the spatial distribution of internal curing water, and the pore structure formed after water release. Consequently, different SAP particle size may lead to different hydration efficiencies and pore characteristics, resulting in varying effects on compressive strength [30,32]. Particularly, the incorporation of 0.3% SAP with a particle size of 180–600 μm resulted in the smallest reduction in compressive strength, with the 180-day compressive strength reaching 137.7 MPa.

### 3.3. Internal Relative Humidity

**Figure 6:**
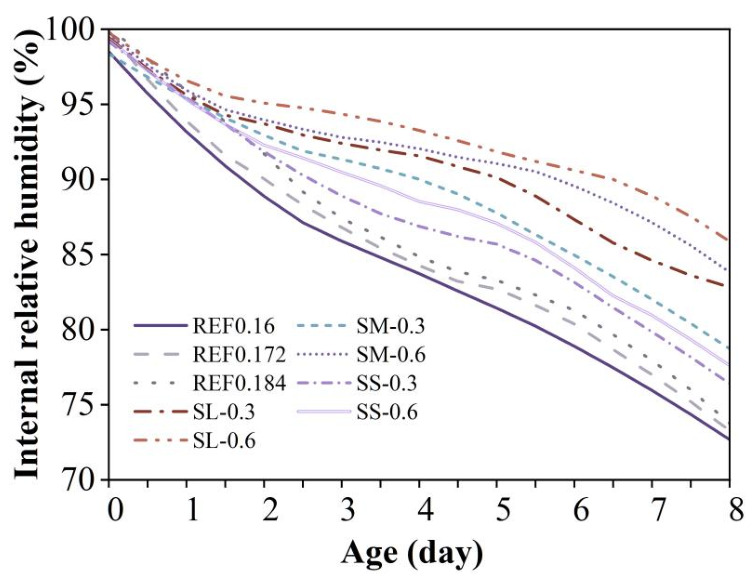
Influence of particle size and dosage of SAP on the internal relative humidity of low-carbon UHPC.

Figure 6 presents the influence of different dosages and particle sizes of SAP on the internal relative humidity of UHPC samples. It is obvious that the internal relative humidity of all UHPC with and without SAP decreased over time. For UHPC without any SAP, the most significant reduction in the internal relative humidity was observed as the hydration progressed. Specifically, the internal relative humidity in control groups REF0.16, REF0.172 and REF0.184 reached only 72.6%, 73.5% and 74.1%, respectively, at the end of testing.

However, after the addition of SAP, the reduction rate of the internal relative humidity in UHPC decreased compared to that of the control groups. This is attributed to the gradual release of water stored in SAP, which compensates for self-desiccation and maintains a higher internal moisture level over time [29,33]. In addition, a higher internal relative humidity can be found in UHPC with increased dosage and particle size of SAP. This can be evidenced by the largest internal relative humidity observed in UHPC SL-0.6 containing 0.6% SAP with the largest particle size range, which reached 85.6% at the same time. These results align with findings from previous studies [14,29], in which the compensation of the internal relative humidity was thought to be attributed by the water released by SAP.

To distinguish the effect of SAP-based internal curing from that of simply increasing the total water content, the SAP-containing mixtures should be compared with the corresponding control mixtures having the same total w/b ratio. Although both mixtures contained the same total amount of water, the SAP-containing mixtures consistently maintained a higher internal relative humidity throughout the curing period. This indicated that the addition water stored in SAP did not behave as free mixing water but was gradually released during hydration to compensate for self-desiccation. In contrast, the additional water in REF0.172 and REF0.184 mainly contributed to the initial mixing process and was rapidly consumed or evaporated during hydration, resulting in only a marginal improvement in internal relative humidity.

### 3.4. Autogenous Shrinkage

**Figure 7:**
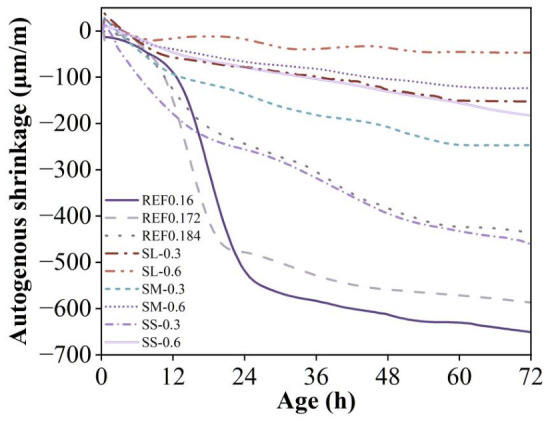
Influence of particle size and dosage of SAP on autogenous shrinkage of low-carbon UHPC.

Figure 7 shows the changes in autogenous shrinkage of UHPC until the 72nd hour after casting. It can be seen from this figure that the autogenous shrinkage behavior of UHPC without SAP can be divided into three stages, especially for the control groups REF0.16 and REF0.172. During the first 12 h, the shrinkage increased slowly, while the rate of autogenous shrinkage gradually accelerated. Subsequently, the shrinkage rate reached a constant value and the autogenous shrinkage steadily increased until the time of 24 h. At last, a stable stage was achieved after 24 h, where the shrinkage rate gradually decreased and the shrinkage curve flattened. At the curing time of 72 h, the autogenous shrinkage values of the control UHPC with w/b ratios of 0.184, 0.172 and 0.16 were 436 με, 587 με and 659 με, respectively. The larger autogenous shrinkage is directly related to the use of silica fume and the extremely low w/b ratio [30]. Moreover, the increase in autogenous shrinkage with reducing w/b is associated with the reduction in internal relative humidity (self-desiccation phenomenon), which induced the increased capillary tension in pore fluids and thus the increased stress on the porous matrix [15].

For UHPC containing SAP, the autogenous shrinkage curve shows a gradual reduction in shrinkage rate over time, as illustrated in Figure 7. It should be noted that compared to the control group REF0.16, the autogenous shrinkage of UHPC containing SAP was significantly reduced. Such mitigation is closely linked to the internal curing provided by SAP, which supplied water during the critical early hydration period, thereby reducing capillary tension and shrinkage strain [9,20]. In order to distinguish the effect of SAP-based internal curing from that of increasing the total water content, the SAP-containing mixtures should also be compared with the corresponding control mixtures having the same total w/b ratio. Despite having identical total w/b ratios, the SAP-containing mixtures exhibited substantially lower autogenous shrinkage than REF0.172 and REF0.184. This comparison demonstrated that the additional water stored in SAP was considerably more effective in mitigating self-desiccation than the same amount of water added directly during mixing.

Notably, for UHPC with higher dosage of SAP, such as SL-0.6 with 0.6% SAP, the autogenous shrinkage decreased by 92.7% at the age of 72 h. In this case, the autogenous shrinkage of UHPC SL-0.6 was almost negligible. At the same dosage, the incorporation of SAP with medium and small particle sizes also exhibited a substantial reduction in autogenous shrinkage by 81.1% and 71.8%, respectively, similar to the findings of other studies [24]. However, at the dosage of 0.3%, the addition of SAP was less effective at reducing autogenous shrinkage, which can be evidenced by the reduction of 76.6%, 62.2% and 29.0% in UHPC SL-0.3, SM-0.3 and SS-0.3. Note that due to the higher water absorption and retention capabilities of SAP with particle sizes of 180–600 μm, UHPC containing larger SAP could maintain higher internal relative humidity by releasing more water and thus the mitigated self-desiccation.

### 3.5. Drying Shrinkage

**Figure 8:**
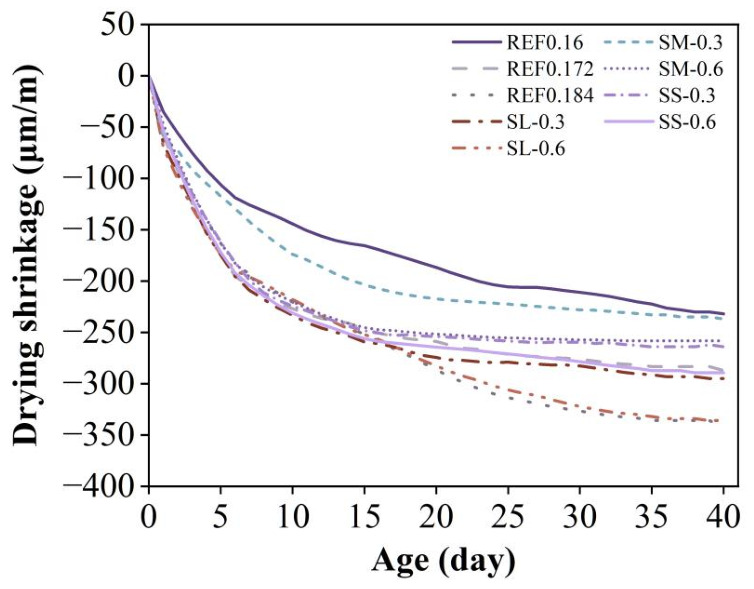
Effect of particle size and dosage of SAP on drying shrinkage of low-carbon UHPC.

The influence of SAP dosage and particle size on the drying shrinkage of UHPC is illustrated in Figure 8. It can be seen from this figure that the drying shrinkage increased by 22.5% and 46.1% for the control groups REF0.172 and REF0.184, respectively, compared with REF0.16. This increase was attributed to the higher w/b ratios, which resulted in greater porosity and a large amount of evaporable water, thereby facilitating moisture loss during drying.

Compared with REF0.16, all UHPC mixtures containing SAP exhibited higher drying shrinkage. This phenomenon can be attributed to the additional SAP-induced voids formed after water release, together with the greater amount of evaporable water stored within the SAP particles, both of which promote moisture loss during drying [23,34,35]. The increase in drying shrinkage was less pronounced for mixtures containing medium- and small-particle SAP than for those containing larger-particle SAP, indicating that SAP particle size also influences the drying shrinkage behavior.

**Figure 9:**
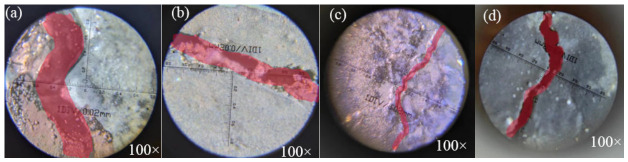
Early-age cracking in UHPC mixtures (**a**) REF0.16, (**b**) REF0.172, (**c**) REF0.184 and (**d**) SS-0.3.

When compared with UHPC mixtures with the same total w/b ratio, the SAP-containing mixtures exhibited lower drying shrinkage than the control mixture, especially REF0.184, as shown in Figure 9. This comparison demonstrated that the additional water stored in SAP did not contribute to drying shrinkage in the same manner as free mixing water. Unlike the control mixtures, where the additional water existed as free mixing water and readily evaporated during drying, the water absorbed by SAP was gradually released during hydration and participated in cement hydration before drying commenced. Consequently, part of the additional water was consumed to form hydration products rather than remaining as evaporable water, resulting in lower drying shrinkage despite the same total w/b ratio.

Moreover, all SAP-containing UHPC mixtures exhibited lower drying shrinkage than the control group REF0.184. This result suggests that the internal curing effect provided by SAP partially compensated for the increase in evaporable water by promoting continued cement hydration and mitigating the development of drying stresses. Consequently, despite the presence of SAP-induced voids, the overall drying shrinkage of SAP-modified UHPC remained lower than that of the control mixture with the highest w/b ratio [35].

### 3.6. Early-Age Cracking

The microscopic cracks observed using a 100× microscope in the control groups and SAP-containing UHPC mixtures are depicted in Figure 9. It should be noted that in this figure, the crack width in the control groups decreased as the w/b ratio increased. This is attributed to the reduction in the autogenous shrinkage, which mitigated the risk of cracking in UHPC plates. Actually, although the drying shrinkage of UHPC during its exposure to wind increased with a higher w/b ratio, its magnitude was smaller than the reduction in autogenous shrinkage. The reduction in crack width with increasing w/b ratio indicated that simply increasing the total water content can partially mitigate early-age cracking by reducing autogenous shrinkage. However, visible cracks were still observed in REF0.172 and REF0.184, suggesting that increasing the total w/b ratio alone was insufficient to effectively prevent cracking under restrained conditions.

**Figure 10:**
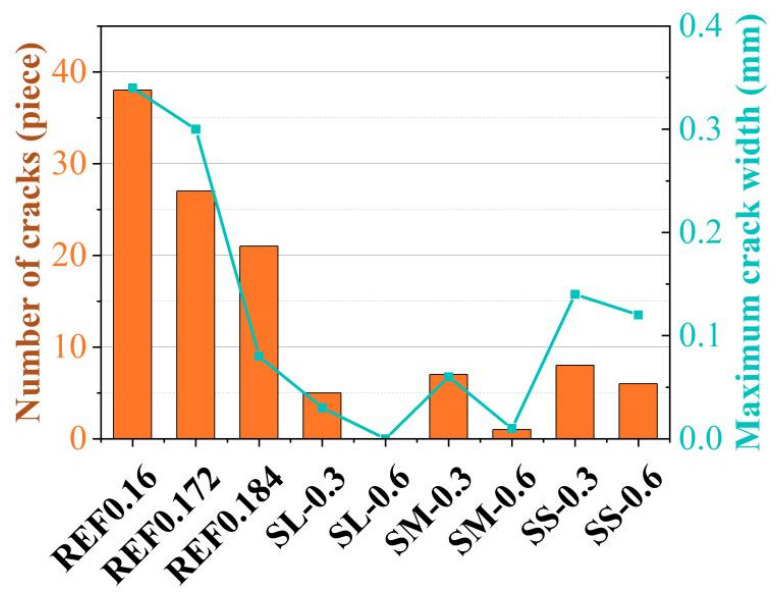
Number of cracks and maximum crack width in UHPC with and without SAP.

**Figure 11:**
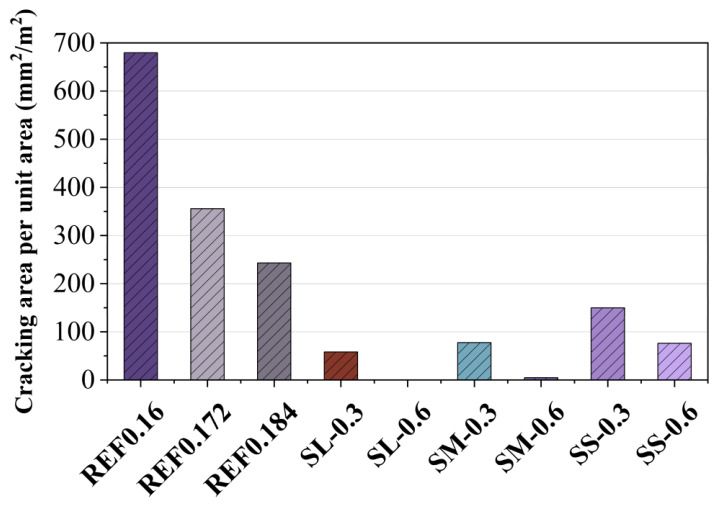
Cracking area per unit area in UHPC with and without SAP.

After the addition of SAP, only micro-cracks ranging from 0.01 to 0.14 mm in the maximum crack width (barely visible to the naked eye) can be observed, indicating a notable inhibition of early cracking in UHPC plates, as shown in Figure 10. Moreover, the number of cracks observed in UHPC containing SAP can also be significantly reduced compared to the control groups. Figure 11 illustrates the crack area per unit area calculated based on the width and length of all cracks in UHPC with and without SAP. It indicates that the addition of SAP significantly suppressed the formation of cracking. Despite having identical total w/b ratios, the SAP-containing mixtures exhibited substantially fewer and narrower cracks than REF0.172 and REF0.184. Specifically, no visible cracks were observed in mixture SL-0.6, whereas cracking still occurred in REF0.184.

### 3.7. XRD

To elucidate the mechanism of SAP-based internal curing, detailed microstructural characterization was carried out on the control mixture (REF0.16) and the representative SAP-containing mixture (SL-0.6), which exhibited the most significant improvement in volume stability. Owing to the large volume of mixtures investigated in this study, comprehensive microstructural analyses were not conducted for all SAP dosages and particle sizes. Therefore, the following results were intended to provide mechanistic insights based on the representative mixture rather than to establish quantitative differences among all mixtures.

**Figure 12:**
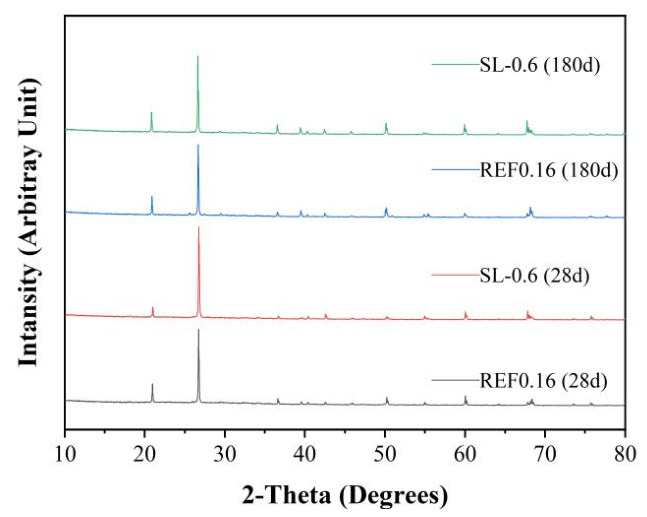
XRD spectra of UHPC with and without SAP at 28 days and 180 days.

The evolution of hydration phases in UHPC with and without SAP at 28 days and 180 days was investigated through the analysis of XRD spectra. As shown in Figure 12, UHPC incorporating SAP exhibited a nearly identical phase composition to the control group REF0.16 at the curing time of 28 days and 180 days. This suggests that the addition of SAP in UHPC did not participate in the formation of any new phases. The detected phases mainly comprised SiO_2_ from aeolian sand, Ca(OH)_2_, CaCO_3_ and unhydrated C_3_S and C_2_S. With an increase in the curing time, a general decreasing trend in the peak intensity of Ca(OH)_2_ was observed, as depicted in Figure 12. The reduction in Ca(OH)_2_ intensity can be attributed to carbonation, during which Ca(OH)_2_ was transformed into calcium carbonate. At equivalent curing ages, UHPC containing SAP exhibited higher Ca(OH)_2_ peak intensities compared to the control group, indicating that the addition of SAP can promote the hydration in UHPC.

### 3.8. TG Analysis

**Figure 13:**
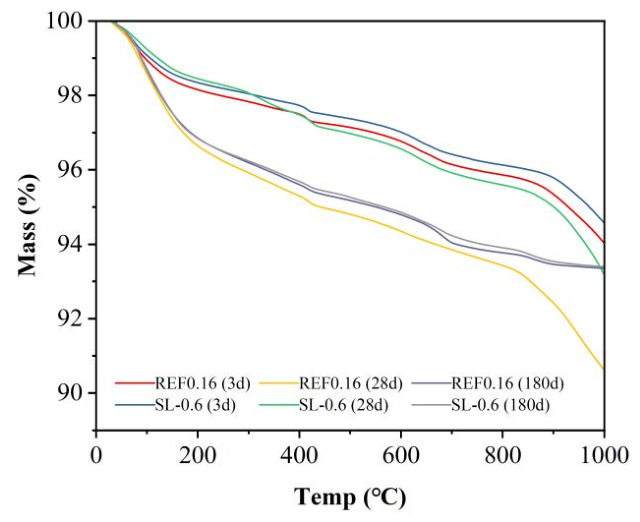
Mass loss of UHPC with and without SAP.

**Figure 14:**
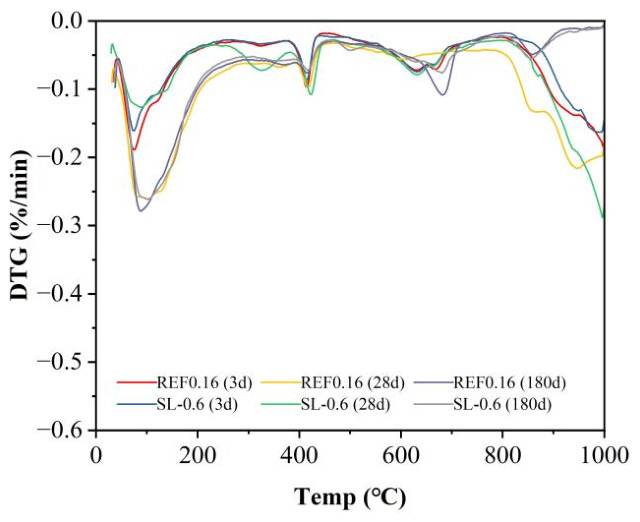
DTG curves of UHPC with and without SAP.

The TG and derivative thermogravimetric (DTG) curves of UHPC samples utilizing different internal curing materials at 3, 28 and 180 days are illustrated in Figure 13 and Figure 14, respectively. It can be seen from these two figures that the major changes in mass in TG curves corresponded to the peaks observed in the DTG curves. Specifically, the mass loss that occurred between 60 °C and 300 °C can be attributed to the loss of water from the interlayer and dehydroxylation of calcium silicate hydrate (C-S-H). Around 420 °C, a peak appeared due to the decomposition of portlandite (Ca(OH)_2_). The decomposition of calcium carbonate can be observed between 600 °C and 750 °C. Peaks above 800 °C were associated with the decomposition of C-S-H, which can result in the formation of tobermorite [36].

**Figure 15:**
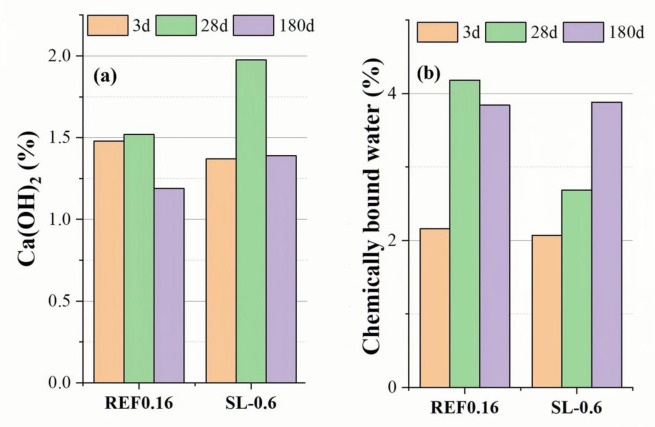
Results of (**a**) Ca(OH)_2_ content and (**b**) chemically bound water in UHPC with and without SAP at various ages.

The content of Ca(OH)_2_ can be quantified using the TG data, as presented in Figure 15a. It can be seen from this figure that UHPC SL-0.6 exhibited a decrease in the content of Ca(OH)_2_ compared to the control group REF0.16 at the curing time of 3 days, which was due to the presence of additional water released from SAP. However, at 28 days, the content of Ca(OH)_2_ in SL-0.6 exceeded that in REF0.16, indicating that the addition of SAP could accelerate the cement hydration by providing extra water. As expected, the content of Ca(OH)_2_ of all UHPC mixtures significantly decreased at 180 days, which was due to the pozzolanic reactions of fly ash and silica fume.

The amount of chemically bound water was calculated based on the mass loss of UHPC between 105 °C and 600 °C [24]. At 28 days, the content of the chemically bound water in UHPC SL-0.6 was lower than that in REF0.16, explaining the reduction in early strength when using SAP. With the extension of the hydration period, by 180 days, the decrease in the degree of hydration was significantly compensated, as the water stored in SAP was released in the later stages, promoting cement hydration.

### 3.9. Pore Structure Analysis

**Figure 16:**
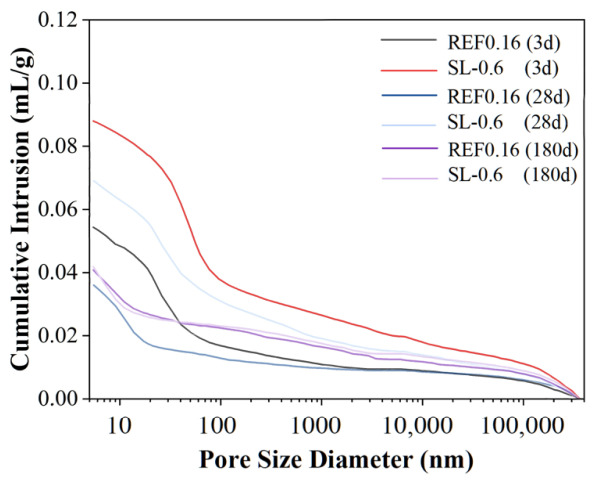
Cumulative pore size distribution of UHPC with and without SAP.

**Figure 17:**
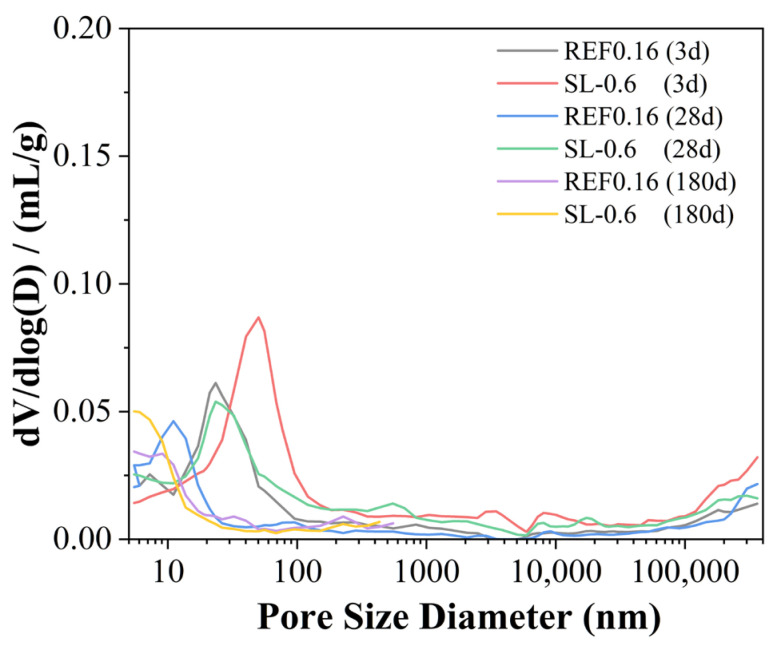
Differential pore size distribution of UHPC with and without SAP.

The development in pore structure of UHPC was revealed by analyzing the cumulative and differential pore size distributions, as shown in Figure 16 and Figure 17, respectively. At 3 days, the critical pore size of UHPC with SAP was larger than that of the control group REF0.16, as presented in Figure 17. With increasing age, the critical pore size of both UHPC mixtures decreased, while the reduction in UHPC containing SAP was greater than that of REF0.16, as shown in Figure 17.

**Figure 18:**
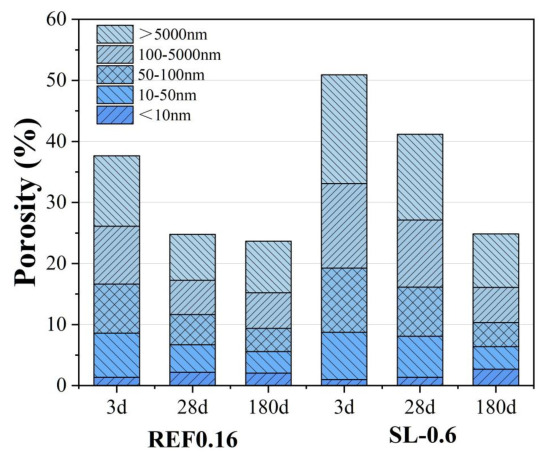
Pore structure characteristics of UHPC with and without SAP.

Based on the MIP data, the total porosity and pore size distribution of UHPC can be obtained, as depicted in Figure 18. It can be observed from this figure that the pore structure of both UHPC mixtures was dominated by pores smaller than 100 nm throughout the curing period. At 3 days, the total porosity of UHPC SL-0.6 was approximately 51%, considerably higher than that of the control group REF0.16 (approximately 38%). This increase was mainly attributed to the larger proportion of pores exceeding 100 nm, particularly those larger than 5000 nm, indicating that the incorporation of SAP initially introduced additional voids into the hardened matrix. The higher early-age porosity was consistent with the lower compressive strength observed for SAP-containing UHPC.

With increasing the curing age, the pore structure of both mixtures gradually became denser. However, the reduction in porosity was much more pronounced for SL-0.6. The total porosity of SL-0.6 decreased from approximately 51% at 3 days to about 25% at 180 days, corresponding to a reduction of 50%, whereas the porosity of REF0.16 decreased from approximately 38% to 24% over the same period. More importantly, the reduction in SL-0.6 was mainly associated with pores larger than 100 nm, while the fractions of finer pores (<100 nm) changed only slightly. These results indicated that SAP primarily refined the coarse pore structure through continued hydration rather than simply reducing the overall pore volume.

The significant refinement of coarse pores can be attributed to the gradual release of water stored in SAP, which promoted continued cement hydration and the formation of additional hydration products, thereby filling capillary pores and densifying the microstructure [37]. However, it should be noted that MIP primarily characterized mercury-accessible pores and may not fully capture closed pores or SAP-generated voids. Therefore, the observed reduction in porosity should be interpreted as evidence of changes in the accessible pore network rather than definitive proof of overall microstructural densification. This interpretation was further supported by the TG results, which showed a continuous increase in the Ca(OH)_2_ content with curing age, indicating the sustained hydration promoted by internal curing. Although no new crystalline phases were detected by XRD, the combined TG and MIP results demonstrated that SAP mainly influenced the hydration degree and pore structure evolution rather than altering the types of hydration products formed [30,35].

### 3.10. SEM and EDS

**Figure 19:**
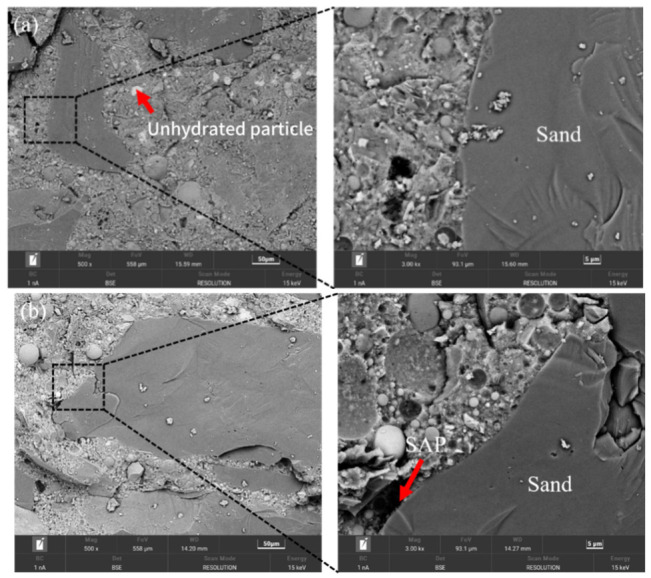
BSE images of (**a**) the control group REF0.16 and (**b**) UHPC SL-0.6 at 28 days.

Figure 19 presents representative backscattered electron (BSE) images of the UHPC microstructure at the curing time of 28 days. It can be seen from Figure 19a that the control group REF0.16 exhibited a relatively dense microstructure with a limited amount of unhydrated cementitious particles. In comparison, several relatively large voids were observed in the SAP-containing mixture (SL-0.6), as illustrated in Figure 19b. These voids were formed after the release of water stored in the SAP particles during hydration and were consistent with higher total porosity and larger proportion of coarse pores quantified by the MIP analysis (Figure 18). The increased early-age porosity provided a reasonable explanation for the lower compressive strength of the SAP-containing UHPC at early ages.

**Figure 20:**
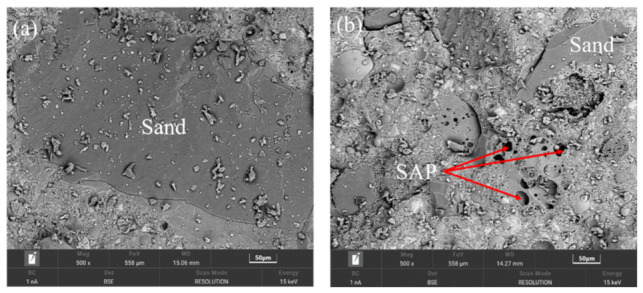
BSE images of (**a**) the control group REF0.16 and (**b**) UHPC SL-0.6 at 180 days.

Representative BSE images of the interface transition zone (ITZ) in UHPC containing SAP at the curing time of 180 days are presented in Figure 20. Compared with REF0.16 (Figure 20a), the ITZ in SL-0.6 (Figure 20b) appeared more compact, with fewer visible interfacial gaps between the cementitious matrix and the aggregate. Although no quantitative image analysis of the ITZ was performed, this observation was consistent with the MIP results, which showed a pronounced reduction in coarse pores between 28 and 180 days, and with the TG results indicating continued hydration under the internal curing effect of SAP. These complementary results suggested that SAP promoted progressive microstructural densification during long-term curing.

**Figure 21:**
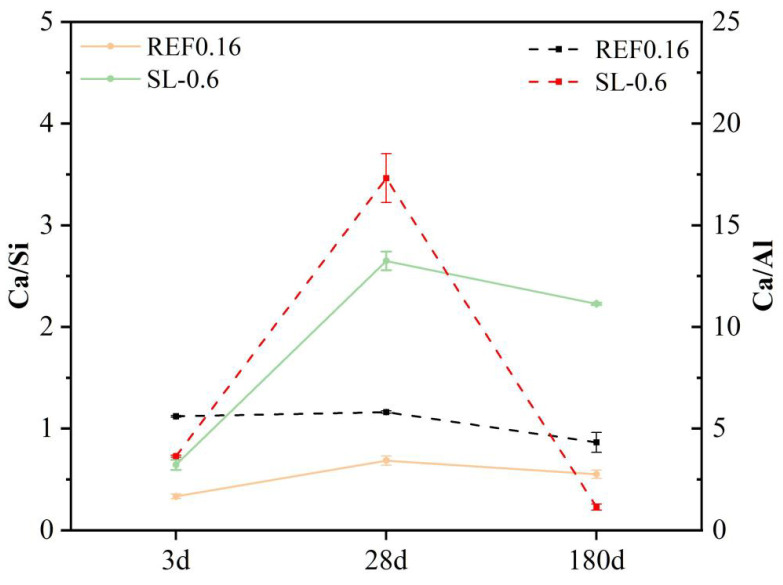
The composition of Ca/Si and Ca/Al of hydration products in UHPC.

The effect of SAP on the hydration products in UHPC was further investigated through EDS analysis, as shown in Figure 21. Note that the data points in this figure represent the average value of Ca/Si and Ca/Al ratios from 35 randomly selected points located within the hydrated cement paste. Unhydrated cement particle, fly ash particles, slag particles, pores and aggregates were excluded from the analysis. It is obvious in Figure 21 that compared with REF0.16, which exhibited relatively stable Ca/Si and Ca/Al ratios throughout the curing period, the SAP-containing mixture showed an increase in both ratios at 28 days, followed by a decrease at 180 days. This evolution was consistent with the continued hydration promoted by internal curing and the progressive pozzolanic reactions of fly ash, slag and silica fume. The later decrease in the Ca/Si ratio suggested the formation of additional calcium aluminum silicate hydrate (C-A-S-H) gel with relatively lower Ca/Si ratio, while the variation in the Ca/Al ratio indicated the participation of alumina released from supplementary cementitious materials (SCM) in the formation of Al-containing hydration products, such as C-A-S-H and AFm phases. These EDS results, together with the TG analysis showing the evolution of Ca(OH)_2_ and the MIP results demonstrating progressive pore refinement, provided complementary evidence that SAP enhanced the hydration process and promoted microstructural densification without changing the crystalline hydration products detected by XRD.

### 3.11. Discussion

Although the incorporation of SAP inevitably led to a slight reduction in compressive strength owing to the formation of SAP-induced voids, the larger particle size effectively minimized this adverse effect while still providing sufficient internal curing. Moreover, as demonstrated by the shrinkage results, this SAP combination significantly improved the volume stability of UHPC by mitigating autogenous shrinkage, reducing early-age cracking, and maintaining high long-term compressive strength. Therefore, from an engineering perspective, the use of 0.3% SAP with a particle size of 180–600 μm provides a balanced compromise between mechanical performance and shrinkage mitigation, making it a promising option for low-carbon UHPC structures where dimensional stability and crack resistance are critical, such as bridge decks, precast elements, and thin-walled structural components.

The adjustment of superplasticizer dosage should also be considered when interpreting the experimental results. However, polycarboxylate superplasticizers mainly improved the dispersion of cement particles and fresh workability, while their influence on long-term hydration products and pore structure was generally limited at a constant effective w/b ratio. Therefore, the differences in mechanical properties, shrinkage behavior, internal relative humidity and microstructure were considered to be primarily associated with the internal curing effect of SAP rather than the slight variation in superplasticizer dosage.

It should be noted that the restrained plate test employed in this study represented an accelerated cracking test rather than a direct simulation of field exposure. The combination of restrained boundary conditions and a constant airflow of 5 m/s was adopted to accelerate moisture evaporation and promote crack initiation within a practical laboratory testing period. Therefore, the test was primarily intended to compare the relative cracking resistance of different UHPC mixtures under identical severe conditions rather than to reproduce actual environmental exposure. Although the drying condition was more severe than that encountered in most engineering structures, the accelerated test provided an effective approach for evaluating the effectiveness of SAP in mitigating restrained shrinkage cracking within a relatively short period.

Although the microstructural analyses were performed only on the representative mixture SL-0.6, the observed mechanisms were consistent with the macroscopic trends obtained for the other SAP-containing UHPC mixtures. Nevertheless, quantitative microstructural comparisons among different SAP dosages and particle sizes require further investigation.

## 4. Conclusions

In this study, low-carbon UHPC was prepared using high-volume fly ash and aeolian sand. SAP was incorporated into the low-carbon UHPC to improve its volume stability by providing internal curing. Based on the experimental results and discussion, the following conclusions can be drawn.

(1) The incorporation of SAP reduced the flowability of UHPC, and the reduction became more pronounced with increasing SAP dosage. At the same dosage, larger SAP particles caused a greater reduction in flowability than smaller particles.

(2) The compressive strength of UHPC containing SAP can be reduced, with the adverse effect becoming more pronounced with higher dosages and smaller particle sizes of SAP. Among the UHPC mixtures investigated, the incorporation of 0.3% SAP with a particle size of 180–600 μm provided the best balance between mechanical performance and shrinkage mitigation, achieving 180-day compressive strength of 137.7 MPa.

(3) The incorporation of SAP provided internal curing for UHPC, effectively mitigating the decrease in internal relative humidity and significantly reducing autogenous shrinkage, thereby inhibiting early-age cracking. Although SAP-containing mixtures showed higher drying shrinkage than REF0.16, their drying shrinkage was lower than that of REF0.184 with the same total w/b ratio. The internal relative humidity at 7 days of UHPC SL-0.6 containing 0.6% SAP was 85.6%. Compared to REF0.16, the autogenous shrinkage of UHPC SL-0.6 decreased by 92.7%, and no cracking occurred during the restrained shrinkage test.

(4) Through the XRD analysis, there were not any new phases detected in UHPC containing SAP at 28 days and 180 days. With increasing the curing time, the peak intensity of Ca(OH)_2_ decreased. Compared with the control mixture, the representative SAP-containing mixture (SL-0.6) exhibited higher Ca(OH)_2_ peak intensities at all curing ages, which was consistent with the continued hydration promoted by internal curing.

(5) For the representative SAP-containing mixture (SL-0.6), MIP results indicated a higher proportion of large pores at early ages but a more pronounced refinement of the accessible pore structure with prolonged curing compared with REF0.16. Combined with TG, SEM, and EDS analyses, these observations suggest that internal curing promoted continued hydration and microstructural development over time. Furthermore, SEM observations indicated a denser ITZ in SL-0.6 at 180 days, while the EDS results were consistent with enhanced pozzolanic reactions.

## Figures and Tables

**Table 1:** Chemical composition of the cementitious materials (%).

Chemical Composition	Cement	Fly Ash	Slag	Silica Fume
SiO_2_	20.60	50.20	34.50	94.56
Fe_2_O_3_	3.22	5.57	1.03	0.07
CaO	63.83	3.95	34.00	0.14
MgO	1.30	1.27	6.01	0.29
Al_2_O_3_	4.84	33.31	17.70	0.09
SO_3_	3.69	0.30	1.64	1.25
K_2_O	0.67	3.10	0.43	0.54
Na_2_O	0.17	0.49	0.25	0.30
P_2_O_5_	0.07	0.05	0.03	0.14
LOI	1.4	1.76	0.13	2.62

LOI: loss on ignition.

**Table 2:** Mix proportions of low-carbon UHPC.

Mix	Cement	Fly Ash	Silica Fume	Slag	Sand	Water Reducer	Total w/b	Effective w/b	SAP
REF0.16	30	50	10	10	100	1	0.16	0.16	0
REF0.172	30	50	10	10	100	1	0.172	0.172	0
REF0.184	30	50	10	10	100	1	0.184	0.184	0
SL-0.3	30	50	10	10	100	1	0.172	0.16	0.3
SL-0.6	30	50	10	10	100	2	0.184	0.16	0.6
SM-0.3	30	50	10	10	100	1	0.172	0.16	0.3
SM-0.6	30	50	10	10	100	2	0.184	0.16	0.6
SS-0.3	30	50	10	10	100	1	0.172	0.16	0.3
SS-0.6	30	50	10	10	100	2	0.184	0.16	0.6

## Data Availability

The original contributions presented in this study are included in the article. Further inquiries can be directed to the corresponding authors.
